# Polyphenols and IUGR Pregnancies: Effects of Maternal Hydroxytyrosol Supplementation on Postnatal Growth, Metabolism and Body Composition of the Offspring

**DOI:** 10.3390/antiox8110535

**Published:** 2019-11-08

**Authors:** Marta Vazquez-Gomez, Ana Heras-Molina, Consolacion Garcia-Contreras, Jose Luis Pesantez-Pacheco, Laura Torres-Rovira, Beatriz Martinez-Fernandez, Jorge Gonzalez, Teresa Encinas, Susana Astiz, Cristina Ovilo, Beatriz Isabel, Antonio Gonzalez-Bulnes

**Affiliations:** 1 Faculty of Veterinary Medicine, UCM, Ciudad Universitaria s/n. 28040 Madrid, Spain; mvgomez@ucm.es (M.V.-G.); tencinas@ucm.es (T.E.); bisabelr@vet.ucm.es (B.I.); 2 SGIT-INIA, Ctra. de La Coruña Km. 7,5. 29040 Madrid, Spain; andelash@ucm.es (A.H.-M.); garcia.consolacion@inia.es (C.G.-C.); jose.pesantez@ucuenca.edu.ec (J.L.P.-P.); torrerovi@gmail.com (L.T.-R.); astiz.susana@inia.es (S.A.); ovilo@inia.es (C.O.); 3 School of Veterinary Medicine and Zootechnics, Faculty of Agricultural Sciences, University of Cuenca, Avda. Doce de Octubre, 010220 Cuenca, Ecuador; 4 Micros Veterinaria, Campus de Vegazana, 24007 Leon, Spain; beatriz@microsvet.es (B.M.-F.); info@microsvet.es (J.G.)

**Keywords:** antioxidants, intrauterine growth restriction, litter-size, pregnancy, swine model

## Abstract

Maternal supplementation with the polyphenol hydroxytyrosol in a swine model of intrauterine growth restriction (IUGR) improves the fetal oxidative status, decreases the appearance of low birth-weight neonates and favors growth during early postnatal stages (lactation). The current study aimed to determine whether hydroxytyrosol supplementation can also improve developmental patterns, metabolic traits, and body composition of the offspring during later postnatal stages (from weaning to adulthood). A total of 21 piglets born from control untreated sows and 20 piglets born from sows treated with hydroxytyrosol during the last two-thirds of pregnancy were selected on the basis of similar body weights at weaning, for avoiding any interfering effects occurred during lactation. The pigs in the treated group had higher average daily weight gain (ADWG) and, therefore, reached higher body weight and corpulence, greater muscle development and higher adiposity than their control counterparts. The following were not found: significant effects on metabolism and body composition except changes in the muscular fatty acid composition of the treated pigs coming from the largest litters; those more affected by IUGR processes. These findings suggest that maternal supplementation with hydroxytyrosol may improve juvenile development of offspring in at-risk pregnancies and pave the way for more specific studies aiming to elucidate effects on adiposity, metabolism, and meat organoleptic characteristics.

## 1. Introduction

In both humans and animal species, Intrauterine Growth Restriction (IUGR) and subsequent Low Birth Weight (LBW) is a common feature in pregnancies challenged by either maternal malnutrition/hypoxia or placental insufficiency [1]. Offspring with LBW is affected by increased perinatal mortality and morbidity in the short-term and by decreased growth patterns and health status and performance in the long-term [2].

Possible strategies for preventing or alleviating the occurrence of IUGR are based on maternal supplementation with antioxidant agents [3], since the IUGR condition is characterized by a weakened antioxidant defense system of the fetuses [4,5]. Polyphenols are potent antioxidant agents and the most abundant dietary antioxidants [6]. Our group has proven the usefulness of maternal supplementation with hydroxytyrosol (a polyphenol present in olive leaves and fruits [7,8] with even higher antioxidant capacity than vitamin E [9]) for improving fetal oxidative status and decreasing the appearance of LBW neonates in a swine model of IUGR [10,11].

Polyphenols are well-considered and freely consumed by the general population because they are perceived as “natural” products for the prevention and treatment of different diseases. In the same way, there is a strong trend for its use during pregnancy and, as an example, 78% of the pregnant women in United States of America take supplements during pregnancy [12]. However, there is a scarcity of data from studies evaluating the practical benefits and potential hazards of polyphenols supplementation during pregnancy [13]. Hence, long-term preclinical studies are necessary to determine the real effects of maternal polyphenol supplementation on the postnatal development and the health/disease status of the offspring. We have previously studied the effects from hydroxytyrosol on prenatal and early postnatal (lactation) development. The current study aimed to increase the available information by determining the effects of the hydroxytyrosol prenatal supplementation on the growth patterns, metabolism and body composition in our swine IUGR model during later stages, during the juvenile development.

## 2. Materials and Methods

### 2.1. Animals and Experimental Procedure

The study was carried out at the INIA animal facilities according to the EU Directive 2010/63/UE for the protection of animals used for scientific purposes. The experimental procedures were specifically assessed and approved by the INIA Committee of Ethics in Animal Research (report CEEA 2013/036).

The experiment involved 93 Iberian piglets born from 18 pregnant sows which, at Day 35 of pregnancy, were pair-matched according to body weight in two equal groups (control and treated with hydroxytyrosol; *n* = 9 each one). These sows were fed with a diet (further data in Table S1) fulfilling 50% of daily maintenance requirements from Day 35 of gestation until delivery [14] to impose a nutritional challenge inducing IUGR in the offspring [15,16]. Nine of them (group HT) acted as the treated group by receiving 1.5 mg of hydroxytyrosol per kg of feed per day while the other nine sows remained as the untreated control group (group C).

After birth, piglets remained with sows until weaning at 25 days-old. At weaning, 41 piglets (10 females and 11 males from the group C and 10 females and 10 males from the group HT), representative of average weight and size of the breed and similar between both groups, were chosen from different litters for avoiding the effects of sow and birth-weight. Currently, it is well-known that weaning-weight has an ineludible effect on postnatal development [17] and, for avoiding masking effects of this variable on the results of the study, we selected piglets with similar weaning-weights. Since the maternal hydroxytyrosol supplementation might improve the development during lactation [10], this design allow properly assessing its effect on postnatal development only after weaning. After weaning, the piglets were fed with two standards diets adapted to age (26–60 and 60–180 days-old; further data in Table S1). The amount of feed offered was re-calculated with age for fulfilling daily maintenance requirements. One female from each group died during the experimental period, so 39 pigs were finally used for the study. In these pigs, we studied the effects of the maternal hydroxytyrosol supplementation on postnatal changes in body weight and size, adiposity, plasma indexes of glucose and lipid metabolism, histological characteristics of muscle and fat, and tissue fatty acid (FA) composition after weaning. From the results obtained analyzing fatty acid composition, the biobanked organs of 52 piglets which were born from the same litters and sampled at weaning (6 females and 11 males from the group C and 10 females and 25 males from the group HT) were used for determining the FA composition.

### 2.2. Evaluation of Growth Patterns, Corpulence and Adiposity during Juvenile Development 

In all pigs, body weight and size (occipito-nasal length, biparietal diameter, trunk length, maximum thoracic diameter, and abdominal and thoracic circumferences) were recorded from weaning to 180 days of age (average of 25, 45, 60, 75, 90 120, 150 and 180 days-old). The body weight values were used to calculate average daily weight gain (ADWG) in the intermediate periods of age and for lifetime. Concomitantly, subcutaneous back fat depth (divided into outer and inner layers and total back fat depth) and *longissimus dorsi* diameter were determined at the P2 point (a point at the right side of the animal located at 4 cm from the midline and transversal to the head of the last rib) using a SonoSite S-Series ultrasound machine with a lineal array probe (5–8 MHz; SonoSite Inc., USA).

### 2.3. Evaluation of Blood Indexes of Carbohydrate and Lipid Metabolism during Juvenile Development

At 120, 150 and 180 days-old, after fasting for approximately 16 h, blood samples were drawn from the orbital sinus using EDTA vacuum tubes (Vacutainer Systems Europe, France). Samples were immediately centrifuged at 1500 g for 15 min, and plasma was separated and stored at −20 ℃ until analysis. Parameters of glucose (glucose and fructosamine) and lipid profile (total cholesterol, high-density lipoprotein cholesterol [HDL-c], low-density lipoprotein cholesterol [LDL-c], and triglycerides) were measured by a clinical chemistry analyzer (Saturno 300 plus, Crony Instruments SRL, Rome, Italy), according to the manufacturer’s instructions.

### 2.4. Evaluation of Organ Weights and Histological Characteristics of Muscle and Fat at 180 Days-Old

The pigs were sequentially euthanized by stunning and exsanguination at 180 days-old, in compliance with RD53/2013 standard procedures. Immediately, the weights of head, carcass and total and individual viscerae (adrenal glands, brain, heart, intestine, kidneys, liver, lungs, pancreas and spleen) were determined. The weight-ratios of carcass and individual viscerae to body weight were also calculated and expressed by percentage.

**Figure 1:**
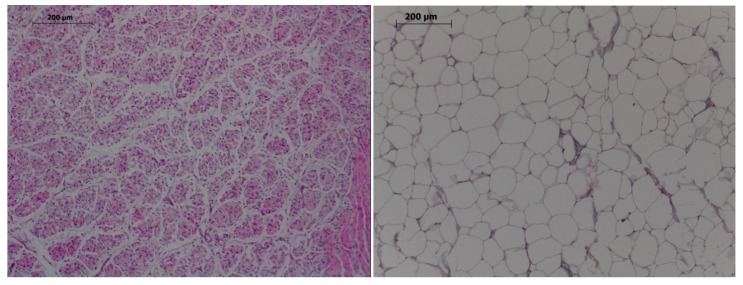
Histological images of muscle (left) and adipocytes (right). Hematoxylin-eosin, 100×, bar 200 µm.

Two samples of muscle (from *longissimus dorsi* at the level of last rib and *gluteus medius*) and subcutaneous back fat were obtained. Immediately, one of them was stored at −20 °C for assessment of total fat percentage and fatty acids composition while the other was fixed in 10% neutral-buffered-formalin and processed for the evaluation of number of muscle fibers and adipocytes after hematoxylin-eosin staining (Figure 1). Total fat percentage and FA composition were also determined in samples from the brain and the right lateral lobe of the liver. One biobanked sample of *longissimus dorsi*, *gluteus medius*, liver and brain from piglets slaughtered at 25 days-old were also used to analyze fatty acid composition.

The third sample of *longissimus dorsi* was used to determine drip-loss capacity on the same day of sampling [18] and meat analysis. For instrumental meat color measures, samples of 2 cm^2^ of area were kept at 4 °C. Color was evaluated on days 0, 3 and 6 postmortem by a spectophotometer (CM-600d, Konika Minolta Sensing Inc., Camera, Japan), previously calibrated with the manufacturer’s instructions. Aperture was set to 10° and light source to D65. The average of three random readings was used to measure lightness (L*), redness (a*), and yellowness (b*) by CIE L*a*b* values and the amount of myoglobin chemical forms was calculated via K/S ratios [19].

### 2.5. Evaluation of Fatty Acid Composition in Diet and Pigs at 25 and 180 Days-Old

Fatty acids in the feed were identified and quantified after extraction and methylation [20], by gas chromatography (HP6890, Hewlett Packard, Avondale, PA, USA) with a capillary column (30 m × 0.32 mm i.d. and 0.25 µm polyethylene glycol-film thickness, HP-Innowax) and a flame ionization detector [21]. The results obtained are detailed in Table S1.

The lipids from intramuscular fat, brain, and liver were extracted (expressed as a dry matter percentage; [22]) and fractionated into the neutral lipid (triglycerides) and polar lipid fractions (phospholipids) [23]. The fatty acids of the subcutaneous back fat were separately analyzed in outer and inner layers. Finally, lipids were methylated and identified [24]. The individual fatty acid percentages were used to calculate proportions of saturated, monounsaturated and polyunsaturated fatty acids (SFA, MUFA and PUFA, respectively), the unsaturation index (UI), and the sum of total ∑n-3 fatty acids and ∑n-6 fatty acids and their ratio (∑n-6/∑n-3 fatty acids) [25]. Moreover the activity of the stearoyl-CoA desaturase enzyme 1 was estimated as desaturation indexes (SCD1; the ratio of the enzyme product, MUFA mainly oleic acid [C18:1n-9], to the enzyme substrate, SFA mainly stearic acid [C18:0]), as previously described [26].

### 2.6. Statistical Analysis

Data were analyzed by the SAS version 9.4 (Statistical Analysis System Institute Inc., USA). First, births were categorized in small (< 8 piglets) and large litters (≥8 piglets), according to Iberian breed data, to analyze the effect of litter size. After testing the normality of data using a Shapiro-Wilk test, dependent variables were assessed using three-way ANOVA in a general linear model including maternal gestational treatment (C vs. HT), sex (female vs. male) and litter size (< 8 vs. ≥ 8 piglets/litter), and all maternal treatment interactions. Dependent variables with changes over time were also assessed by repeated measures ANOVA with the Greenhouse-Geisser correction. Sow was used as a random effect in the weaning analysis to account for the common maternal environment. For performance parameters, the respective age was used as a covariate. Mean values were expressed by estimated marginal means with the different effects included for each variable. The pig was the experimental unit, and the statistical significance was accepted from *p* < 0.05. All the results were expressed as mean ± S.E.M.

## 3. Results

The proper experimental design of the study, based on the selection of piglets with similar body-weight at weaning, caused a lack of significant differences in body weight and corpulence at weaning between groups C and HT. However, even with similar size and weight, the piglets of group HT had a lower diameter of *longissimus dorsi* muscle (10.7 ± 0.7 vs. 9.4 ± 0.6 mm; *p* < 0.05). At that time, the total subcutaneous back fat depth was determined by an interaction between treatment and litter size (*p* < 0.01) and its depth in piglets from small litters in the group HT was higher (5.4 ± 0.6 vs. 3.7 ± 0.5 mm; *p* < 0.01) than in the group C.

### 3.1. Changes in Growth Patterns, Corpulence and Adiposity during Juvenile Development 

**Figure 2:**
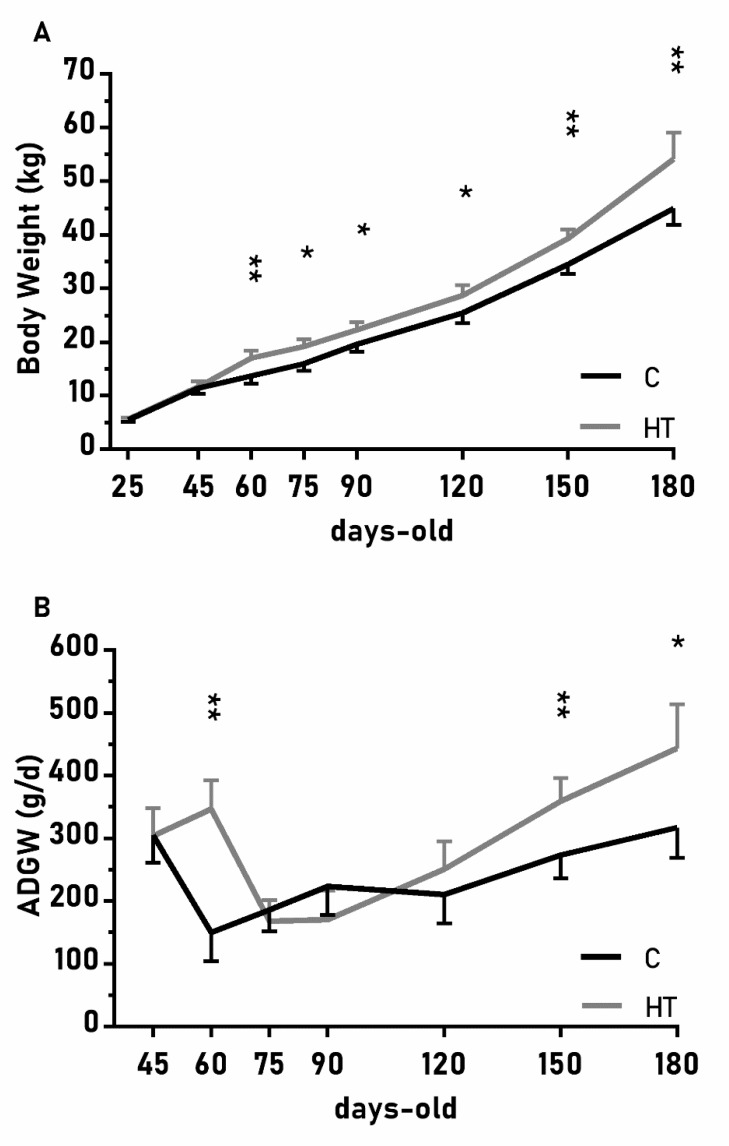
Mean values (± S.E.M.) of (**A**) body weight and (**B**) average daily weight gain (ADWG) from weaning at 25 days-old to 180 days-old in pigs born from sows treated or not with hydroxytyrosol during pregnancy (groups HT, grey line, and C, black line, respectively). Asterisks denote significant differences between groups (*: *p* < 0.05, **: *p* < 0.01).

The changes on body weight from weaning (25 days-old) to 180 days-old were different between groups C and HT (Figure 2A; *p* < 0.01), since the group HT had higher ADWGs and body weight than group C from 60 days-old onwards (*p* < 0.05 for both). The ADWG during the earlier post-weaning phase (25–75 days-old) was affected by an interaction treatment x litter size (*p* < 0.01) and piglets from small litters in the group HT had higher ADWG (329 ± 45 g/d) than the average of groups HT and C (278 ± 23 vs. 221 ± 22 g/d; *p* < 0.01 between them).

**Figure 3:**
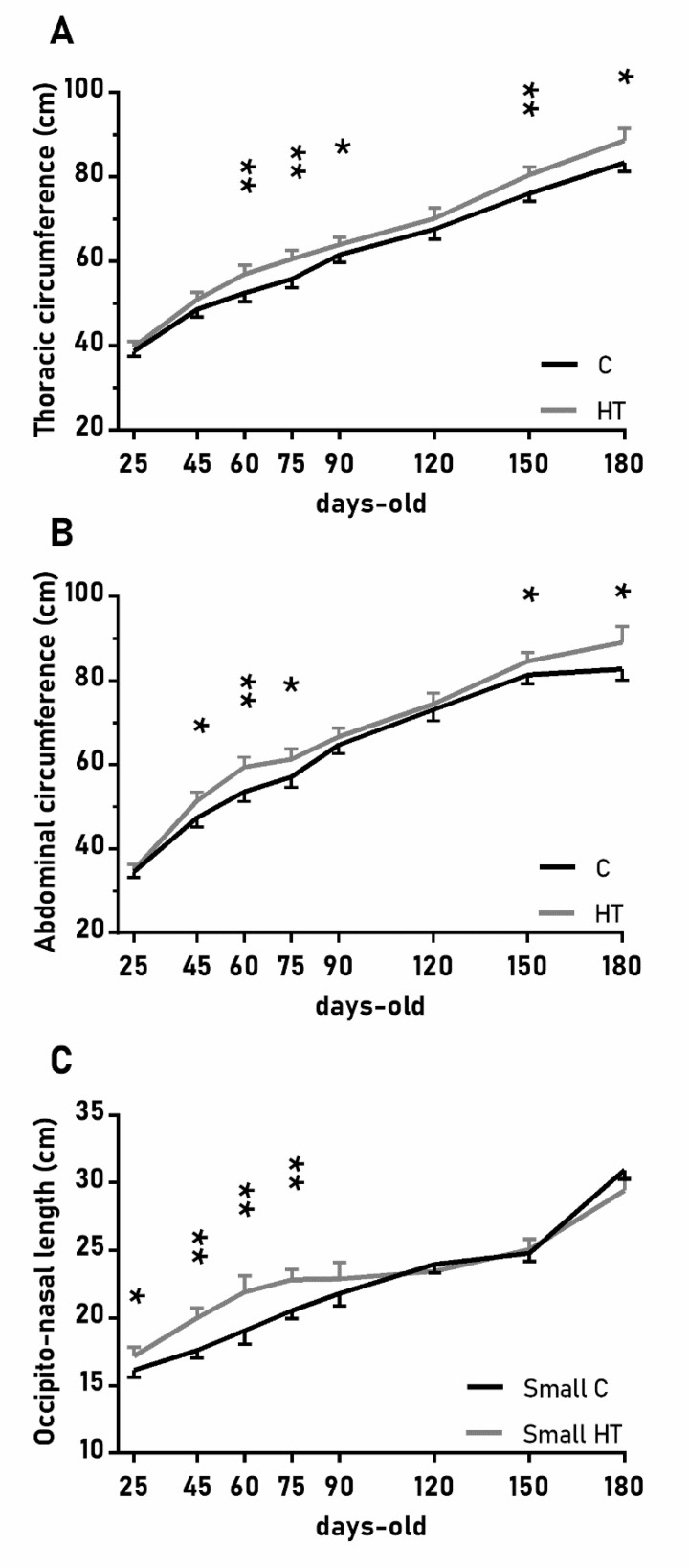
Highlight of significant differences between pigs born from sows treated or not with hydroxytyrosol during pregnancy (groups HT, grey line, and C, black line, respectively) in the mean values (± S.E.M.) of the thoracic and abdominal circumferences respectively (**A**) and (**B**), for pigs from litters of small size (< 8 piglets), the occipito-nasal length (**C**) from weaning at 25 days-old to 180 days-old. Asterisks indicate significant differences between groups (*: *p* < 0.05, **: *p* < 0.01).

The over-time changes in the thoracic and abdominal circumferences were also affected by the treatment (Figure 3A,B; *p* < 0.05 for both) and again values were higher in the group HT than in the group C from 45 to 90 days-old and from 150 to 180 days-old (*p* < 0.05 for both treatments). On the other hand, the effects of the treatment in the head size (occipito-nasal length and biparietal diameter) were modulated by litter size (*p* < 0.05); pigs from small litters in the group HT had longer occipito-nasal length from 25 to 75 days-old than in the group C.

**Figure 4:**
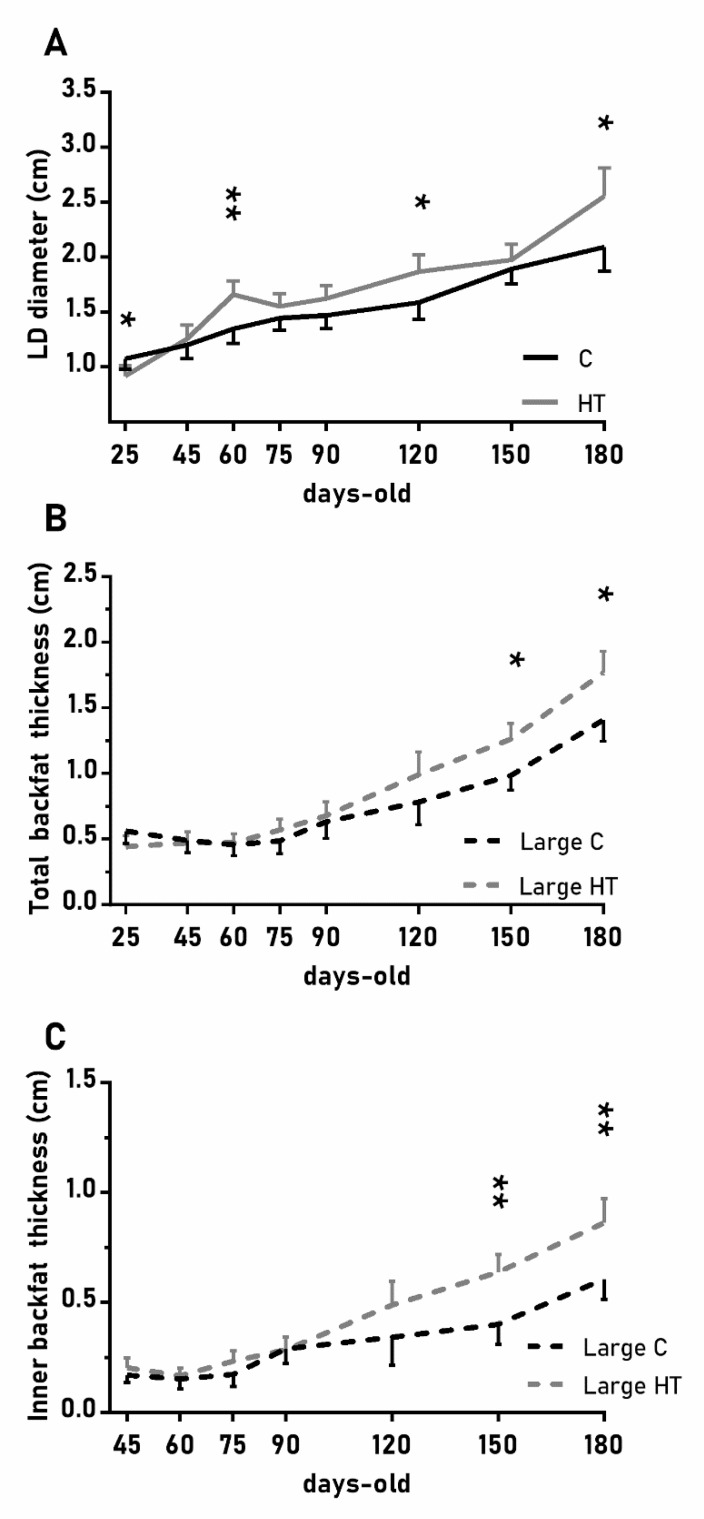
Highlight of significant differences between pigs born from sows treated or not with hydroxytyrosol during pregnancy (groups HT, grey line, and C, black line, respectively) in the mean values (± S.E.M.) of the diameter of the *longissimus dorsi* (LD, panel (**A**) and, for piglets from litters of large size (≥ 8 piglets), the thickness of total and inner-layer back fat (panels (**B**) and (**C**), respectively) from weaning at 25 days-old to 180 days-old. Asterisks indicate significant differences between groups (*: *p <* 0.05, **: *p <* 0.01).

In a similar way, the diameter of the *longissimus dorsi* was also affected by treatment (Figure 4A; *p* < 0.05) and, again, HT pigs showed significantly higher values than C pigs at 60, 120 and 180 days-old (*p* < 0.05 for all). Changes in subcutaneous back fat thickness were determined by an interaction between treatment and litter-size (Figure 4B,C; *p* < 0.01 for total back fat and *p* < 0.05 for the inner layer) with pigs from large litters in the group HT showing greater thickness at 150 and 180 days-old (*p* < 0.05 for both).

Hence, at 180 days-old, the group HT showed a higher body weight (*p* < 0.01) and greater thoracic and abdominal circumferences, diameter of *longissimus dorsi*, and thickness of both total subcutaneous back fat and its inner layer (*p* < 0.05 for all) than the group C (Table 1).

### 3.2. Changes in Blood Indexes of Carbohydrate and Lipid Metabolism during Development

There were no major differences in the plasma concentrations of indexes of carbohydrate and lipid metabolism between treatment groups (further data in Table S2), excepting higher plasma concentrations of fructosamine in the group HT than in the group C at 150 days-old (272 ± 12 µmol/L vs. 253 ± 12 µmol/L, respectively; *p* < 0.05).

### 3.3. Characteristics of Muscle and Fat at 180 Days-Old

There were no significant effects of the treatment on carcass weight and yields (further data in Table S3) or on the absolute or relative weights of viscerae, excepting that the group HT had lower relative weights of liver-to-body (1.9 ± 0.1 vs. 2.1 ± 0.1%; *p* < 0.05) and lung-to-body (0.9 ± 0.1 vs. 1.1 ± 0.1%; *p* < 0.01) than the group C.

Conversely, the group HT showed greater diameter of the *longissimus dorsi* (Table 1; *p* < 0.05) and thickness of total subcutaneous back fat (Table 1; *p* < 0.05) and its inner layer (Table 1; *p* < 0.05). However, there were no differences in the number of adipocytes at the samples of subcutaneous fat. Similarly, there were no differences in the fiber number of both *longissimus dorsi* and *gluteus medius* muscles or in the driploss and color parameters of meat, excepting an interaction between treatment and litter size in lightness on day three after slaughter. Piglets from small litters in the group C had higher lightness than their counterparts in the group HT (58.0 ± 4.4 vs. 47.4 ± 5.6; *p*< 0.05).

### 3.4. Fatty Acid Composition at 180 Days-Old

At 180 days-old, there were found different interactions between treatment and litter size on the fatty acid concentrations of all tissues, excepting the brain (Table 2 and further data in Tables S4–S7).

The assessment of subcutaneous back fat showed a lower content of MUFA and a higher content of SFA, and therefore a lower MUFA/SFA ratio, in the outer layer of the group HT when compared to the group C (Table 2; *p <* 0.01), mainly due to differences in content of C16:0 (Table S6).

The assessment of the diameter of the *longissimus dorsi* showed interactions between treatment and litter size. The fraction of neutral lipids in pigs from large litters in the group HT had a lower content of PUFA and ∑n-6 (*p <* 0.01 for both), mainly due to differences in linoleic acid (C18:2n-6; Table S4). The fraction of polar lipids in small litters of the group HT showed higher ∑n-3 levels and a lower ∑n-6/∑n-3 ratio (Table 2; *p* > 0.01) than the group C, mainly due to differences in docohexaenoic acid (C22:6n-3).

On the other hand, the assessment of the *gluteus medius* showed a lower content of intramuscular fat (*p* > 0.01) and a higher value of moisture in the group HT than in the group C (*p* > 0.05). The neutral lipid fraction of pigs from large litters in the group HT showed lower contents of PUFA and ∑n-6 (Table 3; *p <* 0.05, for both), mainly due to C18:2n-6 (Table S5).

Conversely, the polar lipid fraction of small litters of the group HT showed higher content of PUFA and ∑n-6 and lower MUFA levels and MUFA/SFA ratio than the group C (Table 3; *p <* 0.05 for all), mainly due to differences in arachidonic acid (C20:4n-6; Table S5).

There were fewer differences in the fatty acid composition of viscerae than in muscles. The polar lipid fraction of the liver in pigs from small litters in the group HT showed higher ∑n-3 concentrations and lower ∑n-6/∑n-3 ratio (Table 3; *p* < 0.01 for both) than in the group C. Conversely, large litters showed the opposite effect and the group HT showed lower ∑n-3 concentrations and higher ∑n-6/∑n-3 ratio than the group C (*p <* 0.05 for both). Moreover, the ∑n-6/∑n-3 ratio and ∑n-3 concentrations of the liver neutral lipid fraction showed the same difference patterns in both small and large litters (Table 3).

### 3.5. Fatty Acid Composition at Weaning (25 Days-Old)

At weaning, there were more treatment effects on the fractions of polar lipids of muscles and viscerae than in the fractions of neutral lipids (Tables S8–S11). The group HT showed a higher C18:1/C18:0 ratio in the neutral lipid fraction of *longissimus dorsi* and then the group C (Table 4; *p*< 0.05 for both). The assessment of the polar lipid fraction of *gluteus medius* muscle showed higher MUFA levels and MUFA/SFA ratio in the group HT than in the group C (*p* < 0.05 for both), mainly due to a higher content in oleic acid (C18:1n-9; Table S9). The assessment of the liver showed that the group HT showed higher SFA and lower MUFA concentrations than the group C (Table 4; *p* < 0.05 for both) in the polar lipid fraction, mainly due to C18:1n-9 (Table S11). There was also an interaction with litter size, so piglets from small litters in group HT showed higher ∑n-3 concentrations (6.3 ± 0.8 g/100 g) and lower ∑n-6/∑n-3 ratio (4.8 ± 0.5) in the polar lipid fraction of liver than the piglets from small litters in the group C (∑n-3: 5.1 ± 1.0 g/100g, ∑n-6/∑n-3: 5.8 ± 0.6; *p* < 0.05 for both). The assessment of fatty acids composition of the brain showed that the group HT had lower MUFA concentrations (Table 4; *p* < 0.05) than the group C in the neutral lipid fraction (Table S12).

## 4. Discussion

The results from the present study indicate that a maternal supplementation with hydroxytyrosol during pregnancy has long-lasting effects during the postnatal life of the offspring. In brief, from equal body weight at weaning, pigs from sows treated with hydroxytyrosol had greater growth, body weight, corpulence (wider thoracic and abdominal circumferences), muscle development and adiposity (higher thickness of total and inner layer of subcutaneous back fat) than their control counterparts. Conversely, the main effects on metabolism and body composition were not found, except in changes in the fatty acid composition of pigs from large litters, those more affected by IUGR processes. Hence, the join results of previous research and the current trial suggest that maternal supplementation with hydroxytyrosol improves pre- and early post-natal development of offspring [10] but also juvenile development.

We need to highlight that the data obtained in the present studied were the result of comparing offspring from sows exposed to undernutrition during the pregnancy, either with or without hydroxytyrosol supplementation, without establishing a control group fed *ad libitum* which would allowed to compare the effects of hydroxytyrosol supplementation during restriction with traits in animals without restriction. These obviously is a limitation to the interpretation of the results but the restriction model was established previously and, in the current trial, we preferred to avoid an excessive use of animals in agreement with 3Rs [10,15,16].

It is also highly important to emphasize that the piglets used in the present study had the same body weight in both the control and treated groups at the beginning of the study (at weaning). Afterwards, individuals from pregnancies supplemented with hydroxytyrosol had a higher ADWG and therefore reached a higher body weight than controls as soon as during the early post-weaning phase (25-75 days-old). The development during the post-weaning phases is strongly related to the growth during lactation and afterwards the body weight at weaning [17]; characteristics which have been found to be improved by a maternal hydroxytyrosol supplementation [10]. However, due to the selection of individuals with average and similar body weights, the findings of the present study were independent of the positive effects of prenatal hydroxytyrosol supplementation on birth weight and body development during lactation previously described [10]. Therefore, they could be related to positive effects on physiology and metabolism. There is evidence addressing that the prenatal use of polyphenols may increase epigenetic changes, through DNA methylation, histone modifications and/or variations in the miRNA expression [27,28]. In this sense, a previous study of our group showed that maternal hydroxytyrosol supplementation improved the fetal antioxidant status and prevented possible DNA hypomethylation associated with oxidative stress during the stages concurrent with the maternal treatment [11].

Afterwards, during the last period of the juvenile phase (from 120 to 180 days-old), the maternal hydroxytyrosol supplementation was also found to be related to significant increases in body weight but also corpulence. Increases in body weight and corpulence may be considered a positive effect of the treatment, mainly because of their association with greater muscle development. However, we should also have in mind that they were also related to higher adiposity. First, for pigs and minipigs, thoracic circumference has been found predictive for the amount of carcass fat while abdominal circumference would be predictive for the amount of visceral and subcutaneous fat [29,30,31]. Second, because of a higher thickness of subcutaneous back fat. 

The increase in the subcutaneous back fat depth of the treated group was mainly caused by an increase in its inner layer. The outer layer is more related to protection from cold climates while the inner layer is metabolically more active, mainly due to a high lipoprotein lipase activity [32]. Previous studies have linked the activity of lipoprotein lipase to obesity and insulin resistance [33]; however, there were not found critical changes in the amount of visceral and intramuscular fat or the parameters of glucose and lipid metabolism in the pigs from hydroxytyrosol-treated pregnancies. There were only found higher fructosamine concentrations at 150 days-old, which reflects higher glucose values during the previous weeks. The assessment of fatty acids composition showed that treated pigs had a lower desaturation index in the outer layer of the subcutaneous fat at 180 days-old, which is indicative of a good metabolic status [34,35]. Hence, although we did not found critical changes in metabolism, the evidence of a higher adiposity in the offspring from sows treated with hydroxytyrosol need further consideration and support the necessity of long-term safety data for the use of the compound during pregnancy; having also in mind the possible effects of prenatal polyphenol use on obesogenic gene expression [36].

The results of the current study have a dual value, as a translational animal model for biomedical studies but also for swine production. From the point of view of animal production, the nutritional supplementation of pregnant sows with hydroxytyrosol is highly interesting, since it improves ADWG and growth patterns, giving way to animals with higher body weight and greater muscle development without increasing the weight of internal organs. The assessment of meat quality, in terms of fatty acids composition, showed a lower ∑n-6 content in pigs of large litters from hydroxytyrosol supplemented sows, which may also be useful if considering the human diet recommendations advising to reduce ∑n-6 intake [37,38,39]. Data also supports a better meat quality at earlier life stages, when higher desaturation indexes were also found in the muscles of weaning piglets [40,41,42]. On the other hand, we have to note that, despite a higher diameter of the *longissimus dorsi* in treated pigs, there were no differences in the number of muscle fibers. Hence, the increase in the size of muscle is related to processes of fiber hypertrophy which may mean changes in meat quality traits [43]. Although the present results did not indicate major changes in meat morphological characteristics, such findings deserve further research addressing possible changes in meat organoleptic traits.

Finally, it is important to highlight that, contrary to previous findings in this model [10,11], there were not found any significant effect from offspring sex in growth patterns; such extent may be related with the fact that males were castrated at birth for their management. It is also important to note that pigs from small litters had overall better growth patterns and meat quality characteristics. There were also less pronounced effects from maternal hydroxytyrosol treatment in pigs from small litters than in pigs from large litters. These results are in agreement with previous data addressing a higher incidence of IUGR processes and therefore a higher number of LBW piglets in large litters in both lean and fatty pigs, which compromises postnatal development [10,44,45,46]. However, we need to have in mind that all the piglets in the current experiment had the same weaning body weight, just for avoiding effects of LBW and deficiencies in early postnatal development. Hence, the present trial is suggesting that other effects from litter size may set the basis for further research.

In conclusion, the novel findings of the positive effect of maternal hydroxytyrosol supplementation on postnatal development and meat quality described in the present article show hydroxytyrosol as a useful nutritional tool to improve offspring traits. However, further studies are necessary to elucidate possible effects on adiposity trends and metabolic status, and their real translational value for human beings, and meat characteristics when implemented for improving pork production.

## Figures and Tables

**Table 1:** Mean values (± S.E.M.) of body weight, thoracic and abdominal circumferences, thickness of both total subcutaneous back fat and its inner layer, and diameter of *longissimus dorsi* (LD) in 180 days-old pigs born from sows treated or not with hydroxytyrosol (groups HT and C, respectively). All values are significantly different between treatments (*p* < 0.05 for all).

Variable	Treatment	
	C	HT
Body weight (kg)	44.9 ± 3.0	54.1 ± 4.9
Thoracic circumference (cm)	83.3 ± 2.0	88.6 ± 2.9
Abdominal circumference (cm)	82.8 ± 2.6	89.1 ± 3.7
Total back fat depth (mm)	14.9 ± 1.4	17.6 ± 1.6
Back fat inner layer (mm)	7.3 ± 0.8	9.1 ± 1.0
LD diameter (mm)	20.9 ± 2.1	25.6 ± 2.5

Group C = grupo control. Group HT = treated with hydroxytyrosol.

**Table 2:** Mean values (± S.E.M.) of the fatty acids composition of tissues in 180 days-old pigs born from sows treated or not with hydroxytyrosol (groups HT and C, respectively). All values are significantly different between treatments (*p* < 0.05).

Tissue	Lipid Fraction /Layer	Variable	Treatment	
			C	HT
**GM**	-	Moisture (%)	73.0 ± 2.2	76.8 ± 2.3
	-	IMF (%)	17.2 ± 1.4	13.4 ± 1.5
**Back fat**	Outer	SFA (g/100g FA)	36.3 ± 1.1	38.6 ± 1.2
		MUFA (g/100g FA)	49.7 ± 1.0	47.7 ± 1.0
		MUFA/SFA	1.4 ± 0.1	1.2 ± 0.1

Group C = grupo control. Group HT = treated with hydroxytyrosol. GM = *gluteus medius* muscle. FA = Fatty acids, IMF = Intramuscular fat; SFA = sum of saturated FA, MUFA = sum of monounsaturated FA.

**Table 3:** Mean values (± S.E.M.) of the fatty acids composition of tissues in pigs of small and large litters born from sows treated or not with hydroxytyrosol (groups HT and C, respectively) at 180 days-old. All values are significantly different between treatments (*p* < 0.05).

Tissue	Lipid Fraction	Variable	Groups			
			Small (< 8)		Large (≥ 8)	
			C	HT	C	HT
**LD**	Neutral	PUFA (g/100g FA)	6.1 ± 0.6	6.6 ± 0.8	8.2 ± 0.8	6.5 ± 0.7
		Σn6 (g/100g FA)	5.2 ± 0.5	5.6 ± 0.7	7.1 ± 0.8	5.6 ± 0.7
	Polar	Σn3 (g/100g FA)	3.6 ± 0.2	4.2 ± 0.2	4.1 ± 0.2	4.0 ± 0.2
		Σn6/Σn3	11.8 ± 0.4	10.0 ± 0.6	10.9 ± 0.6	11.2 ± 0.5
**GM**	Neutral	PUFA (g/100g FA)	8.4 ± 1.0	9.2 ± 1.3	11.0 ± 1.1	9.3 ± 0.9
		Σn6 (g/100g FA)	7.4 ± 0.8	8.0 ± 1.2	9.7 ± 1.1	8.1 ± 0.9
	Polar	MUFA (g/100g FA)	16.9 ± 1.0	14.6 ± 1.4	14.1 ± 0.7	14.6 ± 0.6
		PUFA (g/100g FA)	47.6 ± 0.7	49.5 ± 1.0	49.3 ± 0.8	49.3 ± 0.6
		Σn6 (g/100g FA)	43.3 ± 0.6	45.0 ± 0.9	44.9 ± 0.4	44.8 ± 0.8
		MUFA/SFA	0.5 ± 0.05	0.4 ± 0.04	0.4 ± 0.02	0.4 ± 0.02
**Liver**	Neutral	Σn3 (g/100g FA)	2.7 ± 0.4	3.4 ± 0.5	3.6 ± 0.6	2.8 ± 0.5
		Σn6/Σn3	8.3 ± 10.7	6.3 ± 0.9	6.8 ± 0.7	7.8 ± 0.6
	Polar	Σn3 (g/100g FA)	3.6 ± 0.3	4.5 ± 0.5	4.8 ± 0.6	3.8 ± 0.5
		Σn6/Σn3	7.0 ± 0.3	5.5 ± 0.5	5.6 ± 0.5	6.7 ± 0.6

Group C: grupo control. Group HT: treated with hydroxytyrosol. LD = *longissimus dorsi* muscle, GM = *gluteus medius* muscle. FA = Fatty acids, SFA = sum of saturated fatty acids; MUFA = sum of monounsaturated fatty acids, PUFA = sum of polyunsaturated fatty acids.

**Table 4:** Mean values (± S.E.M.) of the fatty acids composition of tissues in pigs born from sows treated or not with hydroxytyrosol (groups HT and C, respectively) at 25 days-old. All values are significantly different between treatments (*P* < 0.05).

Tissue	Lipid Fraction	Variable	Treatment	
			C	HT
**LD**	Neutral	C18:1/C18:0	6.7 ± 0.6	7.8 ± 0.5
**GM**	Neutral	C18:1/C18:0	6.9 ± 0.5	7.7 ± 0.4
	Polar	MUFA (g/100g FA)	19.1 ± 0.6	20.3 ± 0.5
		MUFA/SFA	0.50 ± 0.02	0.54 ± 0.01
**Liver**	Polar	SFA (g/100g FA)	47.8 ± 1.8	51.1 ± 1.2
		MUFA (g/100g FA)	15.2 ± 1.9	11.1 ± 1.4
**Brain**	Neutral	MUFA (g/100g FA)	29.3 ± 0.9	27.5 ± 0.7

Group C = grupo control. Group HT= treated with hydroxytyrosol LD = *longissimus dorsi* muscle, GM = *gluteus medius* muscle. FA = Fatty acids; SFA = sum of saturated fatty acids, MUFA = sum of monounsaturated fatty acids.

## Supplementary Materials

The following are available online at https://www.mdpi.com/2076-3921/8/11/535/s1, Table S1: The composition of diets, Table S2: Indexes of metabolism at 120, 150 and 180 days-old, Table S3: Body composition at 180 days-old, Table S4: The fatty acids profile of *longissimus dorsi* at 180 days-old, Table S5: The fatty acid profile of *gluteus medius* at 180 days-old, Table S6: The fatty acids profile of subcutaneous back fat at 180 days-old, Table S7: The fatty acids profile of liver at 180 days-old, Table S8: The fatty acids profile of *longissimus dorsi* at 25 days-old, Table S9: The fatty acids profile of *gluteus medius* at 25 days-old, Table S10: The fatty acids profile of liver at 25 days-old, Table S11: The fatty acids profile of brain at 25 days-old, Table S12: The fatty acid composition of brain at 25 days-old.
